# MRI-induced artifact by a cochlear implant with a novel magnet system: an experimental cadaver study

**DOI:** 10.1007/s00405-020-06464-z

**Published:** 2020-11-18

**Authors:** Pietro Canzi, Federico Aprile, Anna Simoncelli, Marco Manfrin, Marianna Magnetto, Elvis Lafe, Domenico Minervini, Irene Avato, Simone Terrani, Andrea Scribante, Dzemal Gazibegovic, Marco Benazzo

**Affiliations:** 1 Department of Otorhinolaryngology, University of Pavia, Foundation IRCCS Policlinico “San Matteo”, Viale Camillo Golgi, 19, 27100 Pavia, Italy; 2 Department of Diagnostic Radiology and Interventional Radiology and Neuroradiology, University of Pavia, IRCCS Policlinico San Matteo Foundation, Pavia, Italy; 3 Department of Clinical-Surgical, Diagnostic and Pediatric Sciences, University of Pavia, Pavia, Italy; 4 University of Pavia, Pavia, Italy; 5 Clinical Application Specialist MR/ISP, Philips, The Netherlands; 6 AB GmbH, European Research Centre ERC, Hannover, Germany

**Keywords:** Cochlear implant, Artifacts, Magnetic resonance imaging, Cadaver, Ultra 3D

## Abstract

**Purposes:**

To primarily evaluate MRI-induced effects for Ultra 3D cochlear implantation in human cadavers in terms of artifact generation and MR image quality.

**Methods:**

Three human cadaveric heads were submitted to imaging after unilateral and bilateral cochlear implantation. The 1.5 T MR examination protocol was chosen in accordance with our institutional protocol for the assessment of brain pathology. The maximal signal void size was measured according to each sequence and plane. Two experienced neuro-radiologists and one experienced otoneurosurgeon independently evaluated the MR image quality findings. A 4-point scale was used to describe the diagnostic usefulness of 14 brain structures.

**Results:**

Shape and size of the artifacts were found to be highly related to MRI sequences and acquisition planes. MRI sequences and processing algorithms affected the ability to assess anatomical visibility. Image quality appeared either high or assessable for diagnostic purposes in 9 out of 14 of the ipsilateral structures, in at least one plane. Anatomical structures contralateral to the cochlear implant were highly visible in all conditions. Artifact intrusion clearly improved after application of metal artifact-reduction techniques. In the case of bilateral cochlear implant, a mutual interaction between the two implant magnets produced an additional artifact.

**Conclusions:**

We performed the first cadaver study aimed at systematically evaluating the MRI-induced artifacts produced by a cochlear implant with a novel four bar magnet system. Specific brain structures can be assessable for diagnostic purposes under 1.5 T MRI, with the cochlear implant magnet in place.

## Introduction

The first prototypes of cochlear implants (CIs) appeared no more than 60 years ago and since then, this technology has become a part of common clinical practice, offering perspectives to better life for people suffering from severe deafness [1]. Worldwide, the amount of CI recipients is currently estimated at 400,000 and continuously rises due to the expanding selection criteria [2, 3]. Similarly, in the last decades, the increased advances and availability of magnetic resonance imaging (MRI) have modified the diagnostic–therapeutic approach of substantial percentage of disorders. This has opened a complex debate over CI–MRI compatibility. MRI requests are increasing at approximately 20% per year [2] and an estimated 6% of the population complains of a neurological condition likely to need MRI diagnosis at some point of its lifetime [4]. In CI recipients, MRI presents a number of concerns due to the interaction between the CI’s internal magnet and the complex MR environment: (1) displacement of the entire implant, or of the internal magnet, resulting in pain and device failure; (2) demagnetization or reversal of magnet polarity; and (3) presence of artifacts limiting the diagnostic value of MRI scans. Historically, MRI was considered as an absolute contraindication for CI recipients [5]. With time, MR safety recommendations became “conditional” and a number of solutions were explored by manufacturers to allow MRI for CI recipients [6]. CIs with removable magnets and tight head bandaging represented the historical strategies adopted to reduce the risks of MRI-related CI complications. In 2014, a CI manufacturer (Med-El, Innsbruck, Austria) released a CI model (Synchrony™) utilizing a single magnet, free to rotate in one axis, in order to allow alignment to the stronger magnetic induction field, “just like the needle of a compass” [6, 7]. Minimizing the torque effects on the implant site, this innovation focused on the safety of CIs for up to 3 T MRI machines, without the need of head bandages or magnet removal [2, 8]. Recently, Advanced Bionics AG (AB - Stäfa, Switzerland) released a CI model (HiRes™ Ultra 3D) equipped with a new generation of a magnet system. This innovative magnet design consists of four independent magnet bars, each one able to rotate on its longest axis, mounted in a rotating frame, providing alignment to the magnetic field of an MRI. A first clinical report has documented the MRI effects on the novel Ultra 3D in a patient with residual meningioma, requiring imaging surveillance [9]. More recently, a cadaveric study explored forces and torque of the Ultra 3D, reporting no signs of demagnetization and a reduction of torque in comparison to the standard Ultra CI design [10]. The main objective of this study was to investigate MRI-induced effects for Ultra 3D cochlear implantation in human cadavers in terms of artifact generation and MR image quality. Possible CI displacement due to magnetic field exposure was assessed as secondary objective.

## Materials and methods

### Specimen preparation

Three human cadaveric heads were supplied by the ICLO (San Francis of Sales Foundation, Arezzo, IT) Teaching and Research center. All the donors had offered written informed consent and the specimens were processed in accordance with the current Italian regulations on human body parts for scientific use. The pathological history of the donors was checked to exclude intracranial pathologies or trauma prior to death. All three specimens were fixed in a 5% formaldehyde water solution, using continuous arterial/venous perfusion through the carotid artery and jugular vein. The lowest formalin concentration was employed to minimize a formalin-related MRI artifact [11]. The specimens were maintained in a supine position during the fixing process, to allow the migration of intracranial air bubbles towards the frontal lobes. The specimens were frozen for conservation purposes.

### Surgical procedure

Two Ultra 3D CIs with Slim J electrode arrays were supplied for research purposes. A brief preoperative surgical planning was conducted using skin landmarks. This aimed to reproduce the same positioning of the receiver–stimulator throughout the unilateral and bilateral procedures performed on the three cadaveric heads. A 135° line was identified with respect to the nasion-outer ear canal line and the receiver–stimulator was aligned in this direction, with the center of the internal magnet spaced at 9 cm behind the outer ear canal. This protocol was chosen to reproduce the receiver–stimulator placement that is usually adopted at our institution. A common transmastoid posterior tympanotomy approach was used to obtain electrode array insertion via the round window. All procedures were performed by the same experienced ear surgeon to limit surgical approach variability. The receiver–stimulator was lodged in a sub-periosteal pocket, without bony bed drilling and without the use of fixing sutures. This was to prevent bias for the subsequent CI displacement analysis. A double-layered skin closure was performed at the end of each procedure. The heads were submitted to imaging with the magnet in place. After conclusion of the imaging protocol with a unilateral CI, each cadaveric head was implanted with a contralateral CI, leaving the first CI in place; thus, a bilateral CI imaging protocol was then performed before the heads were finally explanted.

### Imaging study protocol

A Somatom Sensation 64™ (Siemens Healthineers, Erlangen, Germany) scanner was employed for a computed tomography (CT) scan. MRI scans were performed using an Ingenia™ (Philips Medical Systems, Best, Netherlands) 1.5 T MRI scanner. Before implantation with the first CI, baseline CT and 1.5 T MRI scans were conducted to assess the specimen’s eligibility for the study. Potential contraindications included: poor state of preservation of the intracranial structures, presence of metallic foreign bodies such as orthodontic implants or fixtures recognition of possibly unreported intracranial pathology and the presence of a formalin-related artifact. The baseline MRI was also used for comparison, when needed, in the subsequent image quality assessment performed by the neuro-radiologists. After unilateral CI placement, the CT scan was repeated before and after the MRI scan. This same protocol was applied after the bilateral CI placement. A high-resolution protocol was used to obtain CT scans of the entire head with a slice thickness of 0.6 mm. The MR examination protocol was chosen in accordance with our institutional protocol for the assessment of brain pathology, without gadolinium application, focusing on accurate examination of the anatomical areas that were more likely to be obscured by the artifact (e.g., cerebellopontine angle). Each cadaveric head was placed in supine position according to our standard institutional clinical practice. No head wraps were employed to immobilize the internal magnet. The Philips Orthopedic Metal Artifact Reduction (O-MAR) protocol was applied to obtain better image quality [12]. Planar (2D) axial T1-weighted (w) and T2w turbo spin echo (TSE) scans were performed with and without an O-MAR protocol. The acquisition protocol was completed with planar coronal T1w and T2w TSE, volumetric (3D) T1w TSE, volumetric T2w turbo field echo (TFE) sequences and volume isotropic turbo spin echo acquisition (VISTA) sequences. For the MRI scanner, the following parameters were adopted:

- 2D axial T1w: repetition time (TR) 550 ms, echo time (TE) 10 ms, slice thickness 2.5 mm, field of view (FoV) 120 × 179 mm^2^, acquisition time 3:02 min;
- 2D coronal T1w: TR 550 ms, TE 10 ms, slice thickness 2.5 mm, FoV 120 × 179 mm^2^, acquisition time 2:23 min;
- 2D axial T1w with O-MAR protocol: TR550 ms, TE 10 ms, slice thickness2.5 mm, FoV 120 × 179 mm^2^, acquisition time 3:02 min;
- 2D axial T2w: TR 3000 ms, TE 120 ms, slice thickness 3 mm, FoV 150 × 169 mm^2^, acquisition time 4:36 min;
- 2D coronal T2w: TR 3036 ms, TE 120 ms, slice thickness 2.5 mm, FoV 120 × 179 mm^2^, acquisition time 3:51 min;
- 2D axial T2w with O-MAR protocol: TR 6072 ms, TE 120 ms, slice thickness 2.3 mm, FoV 180 × 202 mm^2^, acquisition time 8:42 min;
- VISTA: TR 1500 ms, TE 176 ms, slice thickness 0.8 mm, FoV 150 × 150 mm^2^, acquisition time 5:27 min;
- 3D T1w TSE: TR 600 ms, TE 30 ms, slice thickness 1.1 mm, FoV 260 × 236 mm^2^, acquisition time 7:07 min;
- 3D T2w TFE: TR 2500 ms, TE 278 ms, slice thickness 1 mm, FoV 250 × 250 mm^2^, acquisition time 5:43 min.

### Artifact analysis

**Fig. 1:**
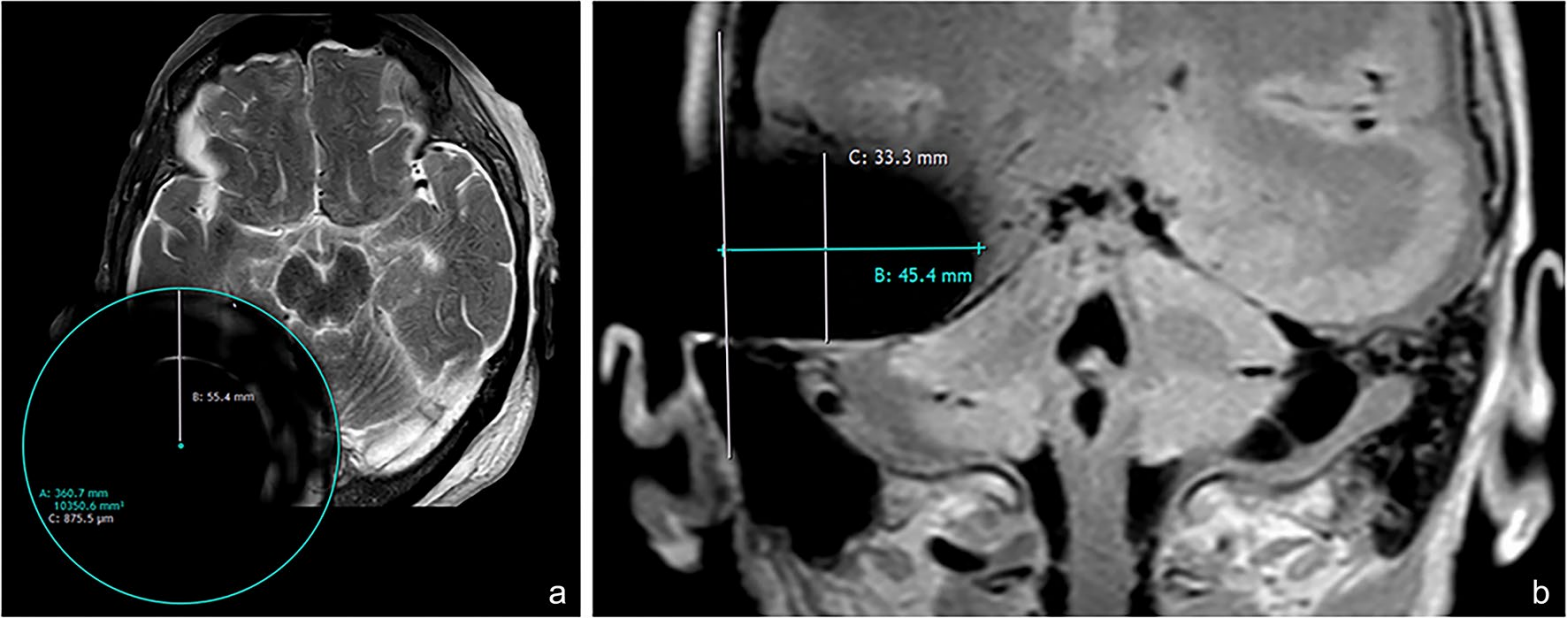
Artifact morphometric analysis. **a** On planar axial sequences, the maximum radius was calculated by matching a circle to the visible edges of the signal void within the brain. **b** On coronal sequences, the maximum latero-medial (45.4 mm in this section) and cranio-caudal (33.3 mm in this section) length were calculated with respect to a vertical landmark passing through the visible portion of the scalp

The maximal signal void size was measured according to each sequence and plane using the IntelliSpace™ Portal (Philips Medical Systems, Best, Netherlands) certified reporting stations. When the planar axial planes were analyzed, a round-shaped artifact was found and the maximum radius was calculated by matching a circle to the visible edges of the signal void within the brain. Since the planar coronal planes produced an oval-shaped artifact, the signal void was described by reporting its maximal latero-medial and cranio-caudal dimensions. A vertical line was drawn tangential to the visible portion of the scalp to define the lateral border of the artifact (Fig. 1a, b). Special care was made to measure the signal void size excluding the signal distortion usually found on the borders of the signal void. Mean length values and standard deviations (SD) of the signal void sizes were calculated.

### Diagnostic usefulness analysis

The CI MRI scans were submitted to two experienced neuro-radiologists and one experienced otoneurosurgeon who each independently evaluated the MR findings. A 4-point scale (0 = completely unusable, 1 = visible but not suitable for diagnostic purposes due to artifact, 2 = obscured by artifact and adequate for diagnostic purposes, 3 = high-quality view of the anatomic structure) was used to describe the following brain structures: frontal lobe, parietal lobe, temporal lobe, occipital lobe, hypophysis, internal auditory canal, cochlea, semicircular canals, vestibulum, brainstem, anterior lobe of the cerebellum, cerebellar vermis, middle cerebellar peduncle and the cerebellopontine angle. The investigation was repeated for each MRI sequence across the three specimens. Ipsilateral and contralateral structures were described in relation to the CI side in case of a unilateral cochlear implantation. When unpaired median structures were investigated (e.g., hypophysis, brainstem, cerebellar vermis), both ipsi- and contralateral sides of each structure were evaluated. Data for the right and left sides were collected in the case of bilateral CIs.

### CI displacement analysis

CT images obtained before and after MRI scans were processed using 3D image rendering post-processing software. Analysis was conducted by strict co-registration of a 3D reconstruction of the skull bones providing the reference point for evaluation of any CI displacement. After co-registration of the skull, the images of the receiver–stimulator package were overlapped and displacement of the CI was estimated (accuracy < 0.5 mm).

### Statistical analysis

Statistical analysis was performed with R software (R version 3.1.3, R Development Core Team, R Foundation for Statistical Computing, Wien, Austria). Descriptive statistics, including the mean, standard deviation, median, minimum and maximum values were calculated for all measures. A Kruskal–Wallis test was applied to determine whether significant differences existed among the measures. The Mann–Whitney test was used as a post hoc measure. Significance for all statistical tests was predetermined at *p* < 0.05. The scores ranged from 0 (completely obscured by the artifact) to 3 (high-quality visualization of the anatomic structure). Diagnostic validity of each structure was determined using the following ranges:

- 0 ≤ *X* < 1.5: not assessable (NA)
- 1.5 ≤ *X* < 2.25: obscured by artifact but assessable for diagnostic purposes (A)
- *X* ≥ 2.25: high quality (HQ)

The 0–0.75 and 0.75–1.5 ranges were grouped together, since they both refer to images that are not useful. Inter-rater reliability was calculated with Pearson’s correlation coefficient.

## Results

### Artifact analysis

**Fig. 2:**
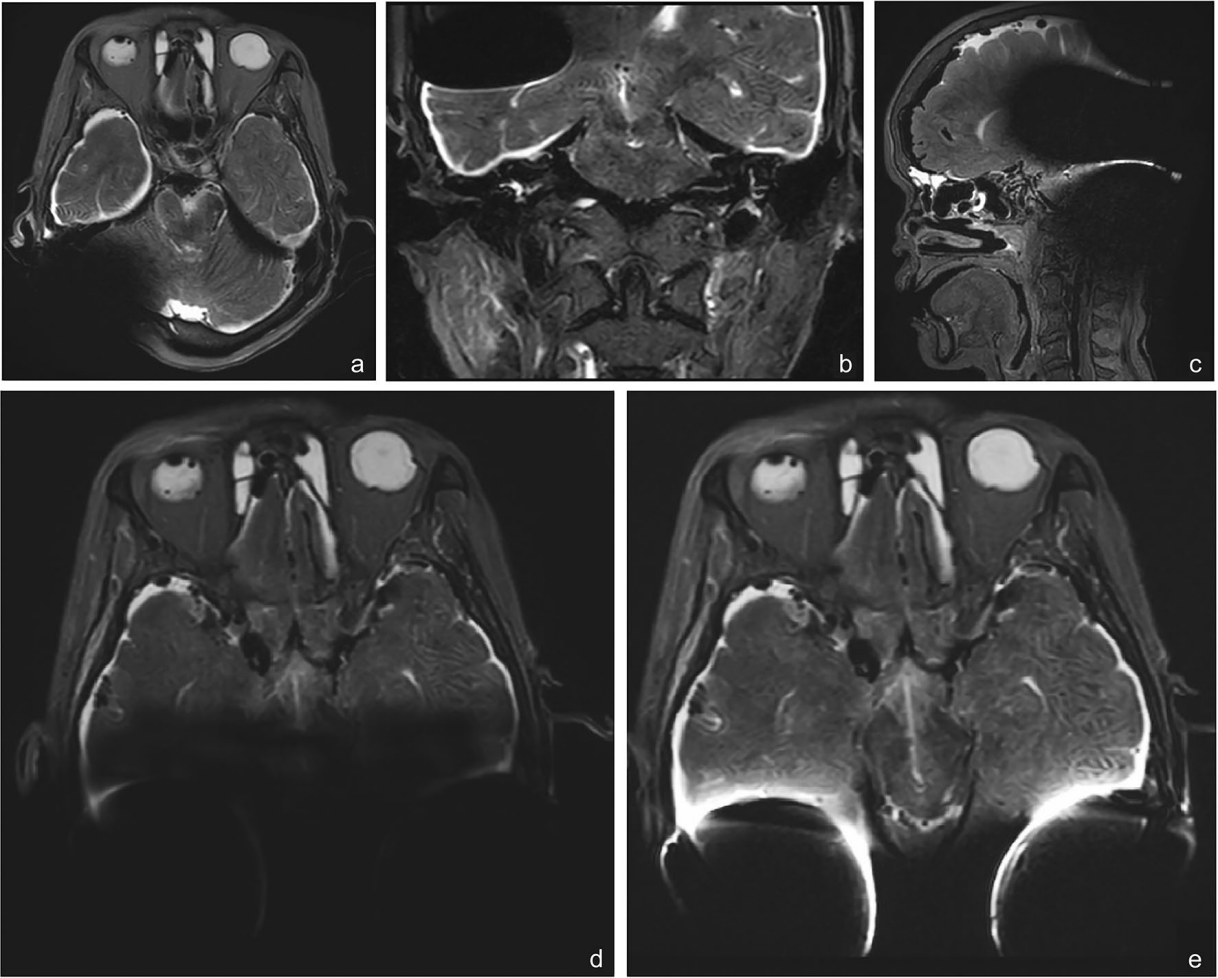
Artifacts analysis in relation to the sequence and plane of acquisition protocols. **a** Round shaped signal void artifact in 2D axial T2w. **b** Oval shaped signal void artifact in 2D coronal T2w. **c** “Shamrock” shaped signal void artifact in 3D T2w TFE. **d** Signal void with bilateral CI: the bilateral mutual artifacts interaction covers anterior structures far beyond the single signal void generated by each CI (2D axial T2w). **e** Bilateral signal void area with O-MAR protocol (same section of **d**)

A signal void area was always found to be centered on the magnet area. Furthermore, signal and geometric image distortion artifacts were observed on the borders of the signal void area. Shape and size of the artifacts were found to be highly related to MRI sequences and acquisition planes. Overall, distinctive round-shaped signal void areas were found in planar TSE sequences acquired for axial planes. Planar TSE sequences acquired for coronal planes returned oval-shaped artifacts. In the case of unilateral CI, the maximum signal void radius was 49.6 mm (SD 7.2) and 56.7 mm (SD 1.8) on planar T1w and T2w TSE axial sequences, respectively (*p* > 0.05). After application of the O-MAR protocol, the radius of the signal void reduced by up to 34.4 mm (SD 10.1) and 36.3 mm (SD 8.4) on T1w and T2w TSE sequences, respectively (*p* < 0.05). Volumetric sequences showed huge and irregular “shamrock-shaped” artifacts, difficult to measure with signal distortion areas larger than 100 mm (Fig. 2a–c). The measurement process made possible only a rough estimate of the maximal radius across of the VISTA sequences that resulted in 62.2 mm (SD 11.7 mm) (*p* > 0.05). For planar T1w and T2w TSE sequences acquired in coronal planes, the signal void size was measured in the latero-medial and cranio-caudal dimensions. The maximal latero-medial length was 59.7 mm (SD 3.8) and 55.4 mm (SD 6.5) for T1w and T2w TSE sequences respectively, (*p* < 0.05). Maximal cranio-caudal length was 37.2 mm (SD 1.7) and 35.1 mm (SD 2.4) on T1w and T2w TSE sequences respectively, (*p* > 0.05). In the case of bilateral CI, a mutual interaction between the two implant magnets produced an additional artifact that was found anteriorly to the signal void area that would be generated by each CI alone (Fig. 2d). Artifact reductions were observed after activation of O-MAR protocol (Fig. 2e). Quantitative measures of the signal void area were not included in this analysis due the irregular shape of the artifacts related to two CIs.

### Diagnostic usefulness analysis

When anatomical visibility was assessed, structures sited contralateral from the CI showed image quality scores higher than all the other conditions tested (*p* < 0.05).

**Fig. 3:**
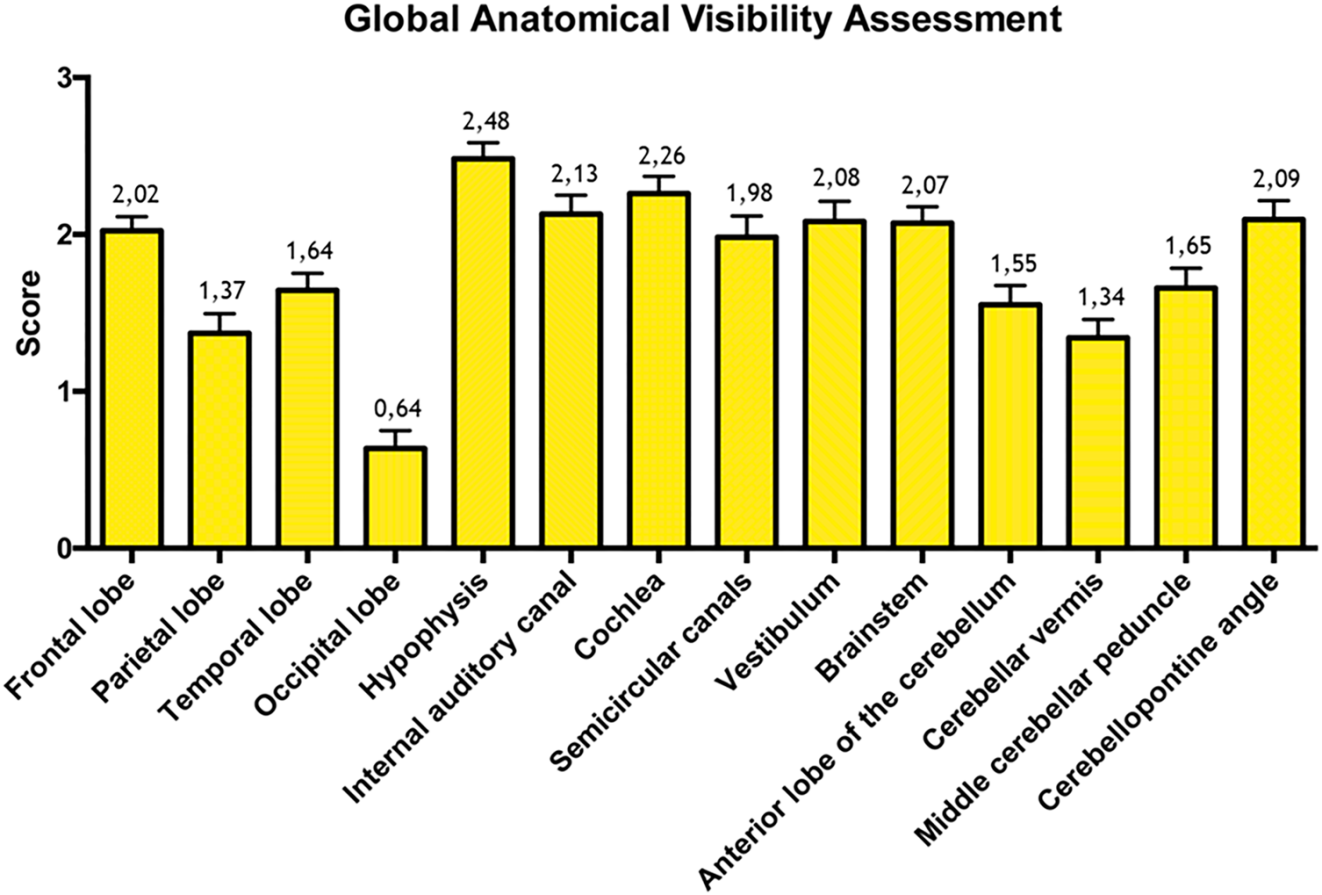
Global anatomical visibility assessment. Global mean scores and confidence intervals resulted from the all CI tested conditions and MRI sequences

**Fig. 4:**
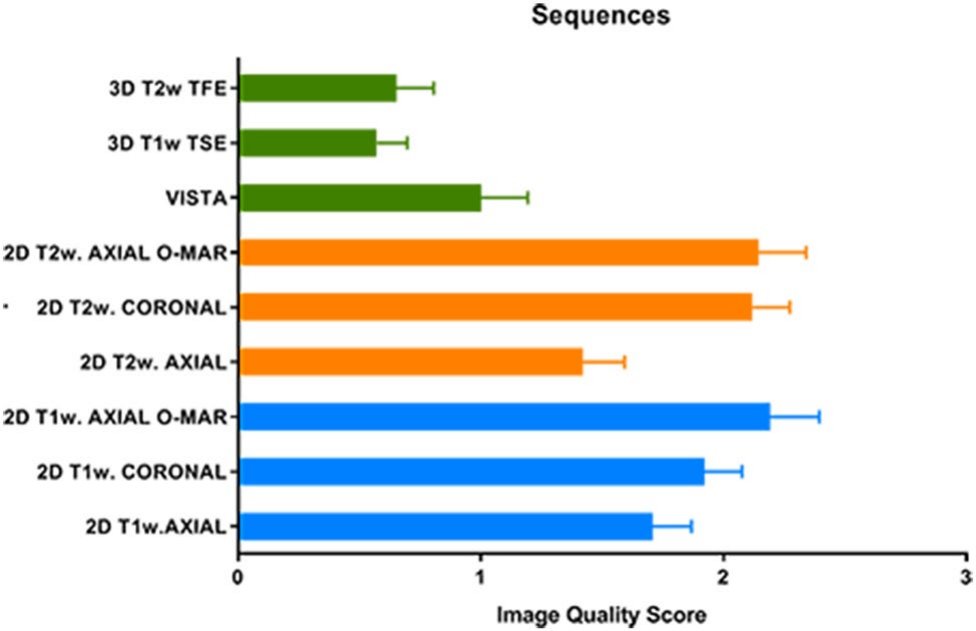
MRI sequences comparison. Mean sequence-specific scores and confidence intervals. Blue rows: 2D T1w sequences; yellow rows: 2D T2w sequences; green rows: 3D sequences. All 2D sequences were obtained with TSE techniques

Except for the occipital lobe and the cerebellar vermis (mean image quality scores of, respectively, 1.80 and 1.83), contralateral anatomic structures returned high-quality diagnostic images (mean score > 2.25), suitable for diagnostic purposes. In the case of bilateral CI, image quality scores were comparable with ipsilateral ones related to the unilateral CI setting (*p* > 0.05). The hypophysis represented an exception, showing lower image quality scores under bilateral CI conditions (*p* < 0.05). Mean scores referred to each CI setting and obtained for all MRI sequences are reported in Table 1. When each anatomical structure was considered, global mean scores across all CI conditions and MRI sequences tested are shown in Fig. 3. The hypophysis achieved the best global mean quality scores (2.48), while the occipital lobe scored the poorest one (0.64). No significant statistical differences were found when the left and right structures were compared in the bilateral CI setting (*p* > 0.05). When each MRI sequence was compared, significant differences were found (Fig. 4). The highest scores were obtained by planar coronal T1w and T2w TSE sequences, planar axial T1w and T2w TSE sequences with the O-MAR protocol applied. No significant differences were found among these (*p* > 0.05). The lowest scores were obtained by 3D sequences (*p* < 0.05). When sequences with and without the O-MAR protocol were compared, O-MAR sequences resulted in better quality outcomes (*p* < 0.05). When planar sequences were compared with volumetric ones, the 2D group showed significantly higher scores for all the anatomical structures investigated (*p* < 0.05) (Table 2). Inter-rater reliability was 0.77, consistent with substantial agreement among the three raters.

**Table 1:** Anatomical visibility assessment: analysis of the diagnostic usefulness under unilateral and bilateral CI condition

Structure	Unilateral CI Contralateral structure (Mean–SD)	Unilateral CI Ipsilateral structure (Mean–SD)	Bilateral CI (Mean–SD)
Frontal lobe	**HQ** (2.77–0.57)	**A** (1.88–0.55)	**A** (1.61–0.69)
Parietal lobe	**HQ** (2.52–0.83)	**NA** (0.96–0.80)	**NA** (1.00–0.77)
Temporal lobe	**HQ** (2.70–0.62)	**NA** (1.25–0.69)	**NA** (1.19–0.65)
Occipital lobe	**A** (1.80–0.55)	**NA** (0.24–0.55)	**NA** (0.13–0.34)
Hypophysis	**HQ** (2.76–0.79)	**HQ** (2.63–0.74)	**A** (2.19–1.02)
Internal auditory canal	**HQ** (2.81–0.53)	**A** (1.92–1.04)	**A** (1.76–1.13)
Cochlea	**HQ** (2.83–0.53)	**A** (2.08–0.91)	**A** (1.95–1.10)
Semicircular canals	**HQ** (2.86–0.52)	**NA** (1.35–1.17)	**NA** (1.32–1.14)
Vestibulum	**HQ** (2.85–0.53)	**A** (1.89–1.14)	**A** (1.61–1.17)
Brainstem	**HQ** (2.48–0.84)	**A** (2.06–0.97)	**A** (1.81–0.81)
Anterior lobe of the cerebellum	**HQ** (2.50–0.80)	**NA** (1.21–0.93)	**NA** (1.11–0.91)
Cerebellar vermis	**A** (1.83–1.08)	**NA** (1.19–1.00)	**NA** (1.11–0.85)
Middle cerebellar peduncle	**HQ** (2.57–0.80)	**NA** (1.31–0.94)	**NA** (1.27–1.01)
Cerebellopontine angle	**HQ** (2.74–0.62)	**A** (1.94–1.05)	**A** (1.69–1.08)

*HQ* high-quality image, *A* image involved by artifact, but assessable for diagnostic purposes, *NA* Non-assessable image, *SD* standard deviation

**Table 2:** Comparison between planar and volumetric sequences

Structure	Unilateral CI setting: ipsilateral anatomical structures		
	2D + 3D sequences (Mean–SD)	2D sequences (Mean–SD)	3D sequences (Mean–SD)
Frontal lobe	A (1.88–0.55)	A (2.16–0.37)	A (1.50–0.51)
Parietal lobe	NA (0.96–0.80)	NA (1.04–0.77)	NA (0.39–0.74)
Temporal lobe	NA (1.25–0.69)	NA (1.47–0.58)	NA (0.75–0.65)
Occipital lobe	NA (0.24–0.55)	NA (0.37–0.66)	NA (0.04–0.19)
Hypophysis	HQ (2.63–0.74)	HQ (2.88–0.33)	A (2.11–1.03)
Internal auditory canal	A (1.92–1.04)	HQ (2.29–0.76)	NA (0.75–1.04)
Cochlea	A (2.08–0.91)	HQ (2.53–0.61)	NA (0.86–0.97)
Semicircular canals	NA (1.35–1.17)	A (2.00–0.94)	NA (0.29–0.66)
Vestibulum	A (1.89–1.14)	HQ (2.25–0.84)	NA (0.54–0.84)
Brainstem	A (2.06–0.97)	HQ (2.63–0.56)	NA (1.21–0.92)
Anterior lobe of the cerebellum	NA (1.21–0.93)	NA (1.49–0.76)	NA (0.64–0.87)
Cerebellar vermis	NA (1.19–1.00)	NA (1.67–0.86)	NA (0.54–0.74)
Middle cerebellar peduncle	NA (1.31–0.94)	A (1.73–0.75)	NA (0.64–0.87)
Cerebellopontine angle	A (1.94–1.05)	HQ (2.29–0.70)	NA (0.68–0.86)

*HQ* high-quality image, *A* image involved by artifact, but assessable for diagnostic purposes, *NA* non-assessable image, *SD* standard deviation

### CI displacement analysis

**Fig. 5:**
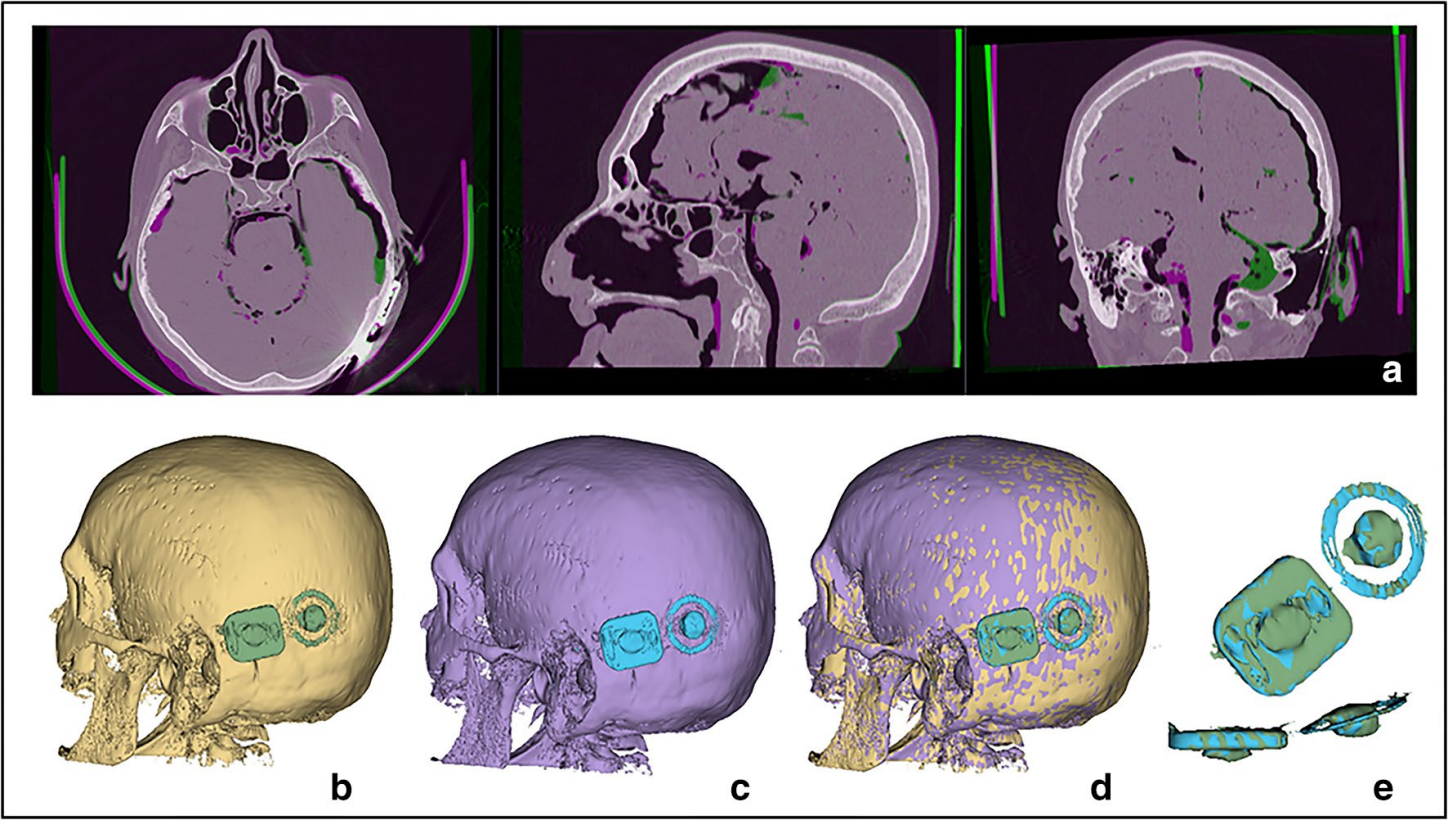
CI displacement analysis. **a** Co-registration of high-resolution CTs before and after MRI, both with CI. Green or purple areas show differences between the two images and are mainly limited to some displacement of soft tissue (brain). Bone structures are rigidly co-registered and do not show any signs of displacement. On the axial view, the device is already visible on both pre- and post- MRI without any signs of repositioning. **b–e** Assessment of CI displacement in 3D reconstruction: **b** 3D head reconstruction before MRI (gray: skull; green: CI), **c** 3D head reconstruction after MRI (purple: skull; cyan: CI), **d** 3D head reconstruction before (gray: skull; green: CI) and after MRI (purple: skull; cyan: CI) to verify potential repositioning: no displacement was recorded, **e** particular of 3D CI reconstruction before (green) and after (cyan) MRI with full CIs concordance

The three-dimensional analysis computed on the basis of the high-resolution CT scans (before vs after MRI), did not report displacement of the CI for any specimen, for either unilateral or bilateral MRI scanning (0 mm for each CI, accuracy < 0.5 mm) (Fig. 5a–e).

## Discussion

As a result of multifactorial interaction, MRI effects essentially depend on the MRI scanning conditions (strength of the magnetic field, MRI acquisition parameters and to some extent also the scanner manufacturer), the CI components (internal magnet and metal parts of the receiver stimulator package) and finally on their mutual influence (magnet position on the skull, angle between the internal magnet and the MRI field). The introduction of adaptive magnets represents the new generation of CIs designed to reduce the limits of MRI–CI interaction [13].Torque forces and demagnetization effects on the new CI with a four-bar magnet system Ultra 3D were previously explored [10]. The aim of the present study was to determine the MRI-induced artifact for human cadavers following implantation with an Ultra 3D implant with the internal magnets in place. Artifacts resulted in signal void areas and distortion signals, both influencing the depiction of the brain’s structures. MRI sequences and processing algorithms affected the ability to assess anatomical visibility: in particular, planar and spin echo sequences resulted in higher image quality in agreement with the published literature [9, 14–16]. Planar T1w and T2w sequences performed equally, as has been previously reported by Crane et al. [17]. Interestingly, MRI scans acquired in different anatomical planes produced different artifacts in terms of signal void and image distortion. Since planar TSE sequences acquired in axial planes returned round-shaped signal void areas, we decided to use the measurement method first described by Cass et al. to provide a more reliable and reproducible signal void assessment [9]. This method, however, was not suitable for describing the oval artifact typical of the coronal acquisition plane, thus a different procedure was adopted. In our study, axial planar T1w and T2w TSE sequences showed a maximum signal void radius of 49.6 mm and 56.7 mm, respectively, in agreement with the artifact radius of 55 mm measured by Cass et al. [9]. Only a few studies have assessed the size of the signal void and moreover, limited mentions on the measurement process have been reported, making difficult a comparison among different CI models. Wagner et al. observed signal voids with an average maximal diameter of 50 mm on cadavers implanted with Synchrony cochlear implant models for 1.5 and 3 T MRI scans [18]. Sharon et al. analyzed one of the largest studies of recipients who received CIs from three different companies: Advanced Bionics, Cochlear Corporation and Med-El. According to the authors, the mean size of the artifact was 46 mm in length and 36 mm in width across all axial sequences for a 1.5 T field strength [14]. Kim et al. described a mean artifact size of 74.3 mm along the long axis and of 41.6 mm along the short axis in 18 CI recipients (3 Med-El, 5 Advanced Bionics and 10 Cochlear Corporation implants) for 1.5 and 3 T MRI scans [19]. With everything considered, the most relevant concern was the diagnostic usefulness of the MRI findings. In our study, the effect of distortion artifacts was investigated for 14 anatomical structures, with the aim of elaborating a systematic description of the brain. The quite high inter-rater agreement among the three blinded observers supported the reliability of our findings. Image quality appeared either high or assessable for diagnostic purposes in 9 out of 14 of the ipsilateral structures, in at least one plane. Coronal plane views resulted significantly better visibility than the axial ones. This is in alignment with other clinical studies [14, 15]. Looking at the ipsilateral posterior fossa anatomy, internal auditory canal, inner ear and cerebellopontine angle, these were found to be partially obscured by artifact but considered acceptable in the best quality MRI sequences. These findings were consistent with previous studies performed on different CI models [9, 14, 15, 17, 20]. Five anatomical areas (parietal lobe, temporal lobe, occipital lobe, cerebellar vermis and the anterior lobe of the cerebellum) were not assessable when ipsilateral to the CI, regardless of the acquisition strategy. In the case of bilateral CI, poorly investigated before [14], the mutual interaction of the two CI magnets produced artifacts involving all anterior and median structures, of particular significance for the hypophysis. To the best of our knowledge, this is the first description of the mutual interaction artifact. These findings may play a significant role for bilateral CI recipients needing MRI surveillance. Previously, only two cadaveric studies aimed at comprehensively describing the brain in relation to the MRI-induced artifacts [18, 21]. Only a qualitative comparison was practicable because no mention of the structures and sequence scores was made by other authors. In agreement with published literature, anatomical structures contralateral to the CI were highly visible in all conditions. However, ipsilateral visibility was only partially consistent to those reported by other studies. This may be due to a number of possible variables such as: the employment of scanners produced by other manufacturers, differences in the acquisition parameters, or sequences and different CI models. Moreover, previous literature has not provided details on the surgical placement of the CI on the skull, something that may influence the final outcome [16]. Remarkably, artifact clearly improved after application of metal artifact-reduction techniques. O-MAR activation significantly increased the image quality, reducing the areas subject to distortion and limiting the signal void radius up to a maximum of about 20 mm. Metal artifact-reduction algorithms were specially developed for better handling of artifacts derived from orthopedic (non-ferromagnetic) implants [6]. However, to the best of our knowledge, no previous work has explored the effect of an O-MAR protocol on CI recipients.

As a second goal, we studied possible CI displacement related to the magnetic field exposure during MRI scanning. Even though no particular mechanical steps were taken to stabilize the implant receiver (e.g., headbands, CI fixing within a drilled bony bed, sutures), the accurate co-registration of CTs before and after MRI imaging revealed the absence of any CI migration. Although CI displacements have not been measured before by means of a co-registration CT system and without the adoption of fixing strategies, our outcomes were aligned with clinical data reported for a freely rotatable magnet system [7] and differently from other internal magnet models [22].

Finally, some restrictions concern the present study. First, MRI-induced artifacts were not investigated for a magnetic field strength higher than 1.5 T (e.g., 3 T). Second, the experimental nature of the study represents a limitation relative to the sample size and the cadaver conservation techniques required. On the other hand, the high statistical significance of the results supports the relevance of our findings, which may provide useful information for clinical practice.

## Conclusions

The consistent advances of both CI and magnetic resonance technologies are progressively making it possible to overcome an historical incompatibility between these. We performed the first cadaver study aimed at systematically evaluating the MRI-induced artifacts produced by a CI with a novel four-bar magnet system. MRI sequences and acquisition planes influence artifact production and, therefore, the ability to assess various anatomical structures. The adoption of specific metal artifact-reduction algorithms may represent a step forward to improve the MR image quality. Clinical studies are mandatory to validate the evidence from our experimental findings.
